# Curve forming prediction of coal mine roadway based on RBF interpolation

**DOI:** 10.1371/journal.pone.0288753

**Published:** 2023-07-21

**Authors:** Zhixiang LIU, Kang ZOU, Zhan SUN

**Affiliations:** 1 Research Institute of Mineral Resources Development and Utilization Technology and Equipment, Liaoning Technical University, Liaoning Province, China; 2 School of Mechanic Engineering, Liaoning Technical University, Liaoning Province, China; Southwest Jiaotong University, CHINA

## Abstract

Underground roadway excavation is a complex process, especially roadway curved excavation. In addition, the rationality of the design of coal mine roadway excavation scheme directly affects the speed of roadway excavation. The more reasonable the scheme design, the more conducive to rapid excavation. In order to avoid the influence of invalid construction on the efficiency of roadway excavation, this paper studies the forming of roadway bend. Based on the analysis of the tunneling process of the roadway curve, the mathematical model of the roadway curve is established. Taking the turning radius of the roadway curve as the evaluation index, the influence of various factors on the roadway curve excavation is analyzed. The research shows that the radius of the roadway curve increases with the increase of the feed rate, the working space position of the roadheader and the required width of the roadway, and decreases with the increase of the working space angle. Then, combined with the advantages of KNN algorithm, an interpolation model for calculating the radius of the curve is established based on RBF algorithm, and the radius of the tunnel curve is reconstructed and predicted. It provides a basis for the rational design of the construction process of the roadway bend and a reliable numerical algorithm for the design of the radius of the roadway bend. It also provides a theoretical basis for improving the efficiency of high roadway excavation in coal mines.

## 1 Introduction

Due to the increasing demand for coal resources and mining intensity, more and more coal mines begin to carry out deep mining [1, 2]. As an indispensable part of coal mining, roadway excavation has always been an important research topic. Coal mine roadway excavation is an important work in underground engineering, which is usually used in mining, coal mine ventilation, transportation and other fields. The process of roadway excavation includes many links such as developing roadway, supporting roadway, drainage and ventilation [3], among which the excavation of roadway bend is more complicated and difficult.

At present, the main problems faced by roadway excavation are: the stability of roadway is still improved, the degree of intelligence and automation of excavation system is not high, the efficiency of roadway excavation is low, and the problem of mechanized mining is out of balance [4–8]. In view of this series of problems, domestic and foreign scholars have done a lot of research: In Reference [9], aiming at the problem of roadway instability, based on the study of actual cases, the numerical simulation method was used to study the failure mechanism of roadway and the new support design, which made a certain contribution to guiding how to improve the stability of roadway. Practice shows that there are ’ cross joints ’ in surrounding rock, and ’ cross joints ’ have an important influence on the stability of unsupported roof. In response to this phenomenon, Reference [10] proposed a cross-joint trace detection algorithm based on image recognition technology, which provides a new means for suggesting the stability of roadway coal rock. In order to alleviate the imbalance of roadway excavation, improve the efficiency of roadway excavation and reduce the cost of generation, a large number of scholars have studied the factors that limit the speed of roadway excavation through field investigation and field analysis, and put forward corresponding solutions to solve practical problems [11–14].

Based on the comprehensive field investigation and the research results of a large number of scholars, the main factors affecting the rapid excavation of roadway are: complex geological structure, low intelligent level of mining machinery, unreasonable excavation scheme and process design, complex transportation system, cutting drilling technology is not advanced enough, operation and maintenance management mode confusion and so on [15]. In the research of advanced cutting technology, Reference [16] proposed that the research on the self-setting directional cutting technology of tunneling equipment can not only effectively improve the intelligent level of tunneling equipment, but also improve the tunneling efficiency and reduce the line error of tunneling roadway. In terms of improving the support efficiency of roadway tunneling, in order to meet the support requirements in the tunneling process of Tunnel Boring Machines (TBMs), Reference [17] compared and analyzed various support methods. Based on the analysis results, the optimization of traditional support methods is realized, which provides a basis for improving support efficiency. In the field of equipment transportation, mine digital operation and maintenance management and intelligent tunneling equipment research and development: Based on the analysis of the problems faced by the navigation and positioning technology of the cantilever roadheader, Reference [18] summarized the development direction of the roadheader patrol transportation system. Yang Jianjian et al. of China University of Mining and Technology have deeply studied the independent perception and control technology of intelligent rapid excavation in fully mechanized excavation face of coal mine, and designed the intelligent mine operating system, which provides a technical basis for improving the excavation efficiency and realizing the integration of management and control [19, 20]. Reference [21] made a comprehensive review of the Roadheader, and defined the important parameters of the tunneling efficiency of the Roadheader. The risk of tunneling equipment deployment is analyzed, and it also provides a theoretical basis for improving the design of roadheader.

Summarizing the research results of many experts and scholars, it can be seen that the current research on how to improve the efficiency of roadway excavation is mostly in the process of roadway excavation operation and maintenance management, excavation methods, and excavation machinery. There are relatively few studies on roadway excavation design. The engineering practice tells us that the rationality of the design of roadway excavation process before roadway excavation has an important influence on the efficiency of roadway excavation. The more reasonable the roadway design is, the more beneficial it is to improve the efficiency of roadway construction. In order to avoid ineffective construction and improve the efficiency of roadway excavation, this paper studies the bending forming.

Based on the analysis of the tunneling process of the roadway curve, the tunneling process of the roadway curve is described. Based on this, a mathematical model of roadway bend considering the influence of working condition error is established. Taking the radius of the roadway curve as the evaluation index, the influence of various factors on the roadway curve excavation is analyzed. In addition, in order to facilitate engineers to quickly make a choice of the scheme, the radius of the roadway curve is interpolated and predicted.

## 2 Roadway bending forming process

The construction methods of roadway curved tunneling include drilling blasting method, mechanical or hydraulic tunneling method [22]. The main processes of drilling blasting method include drilling, charging, blasting, ventilation, rock loading, support and so on. There are many kinds of curve forming methods, the common ones are: The first method is to use the extended string to make a curve ruler, and arrange the blastholes on both sides of the roadway according to the marks on the ruler to make the roadway move forward along the design curve. This method needs to design the curve first, then make the curve scale according to the design requirements, set the blasting point according to the mark on the scale, and finally carry out drilling blasting to make the roadway forming. This method is suitable for simple curves. The second method is to use the theodolite to mark the center line, and determine the radius of curvature and the length of the curve of the roadway by measuring the angle and distance on the center line of the roadway. This method requires accurate measurement and calculation, and can adapt to more complex curve conditions. The advantage of this method is that it can ensure the accuracy and stability of the curve. The third method is to use the level to mark the waist line, and determine the radius of curvature and the length of the curve of the roadway by measuring the elevation difference on both sides of the roadway. This method does not require angle measurement, which is relatively simple and convenient, but it needs to ensure the accuracy of the level. The fourth way is to directly use roadway excavation machinery. This tunneling method is relatively fast, and it is suitable for tunneling roadway curves under various working conditions.

No matter which method is used to excavate the roadway, it is indispensable to support the roadway. Common support methods include timber support, metal support, reinforced concrete support, anchor spray support, etc [23]. The purpose of support is to ensure the stability and safety of roadway and prevent roadway collapse or other accidents. In general, the excavation and forming of the roadway bend requires a variety of methods and techniques. According to the geological conditions and the actual situation, selecting the appropriate tunneling method and forming method can improve the efficiency and quality of tunneling, and further ensure the stability and safety of roadway. In addition, roadway excavation also needs to strictly abide by the working procedures and safety operation regulations to ensure the safety of roadway excavation.

**Fig 1:**
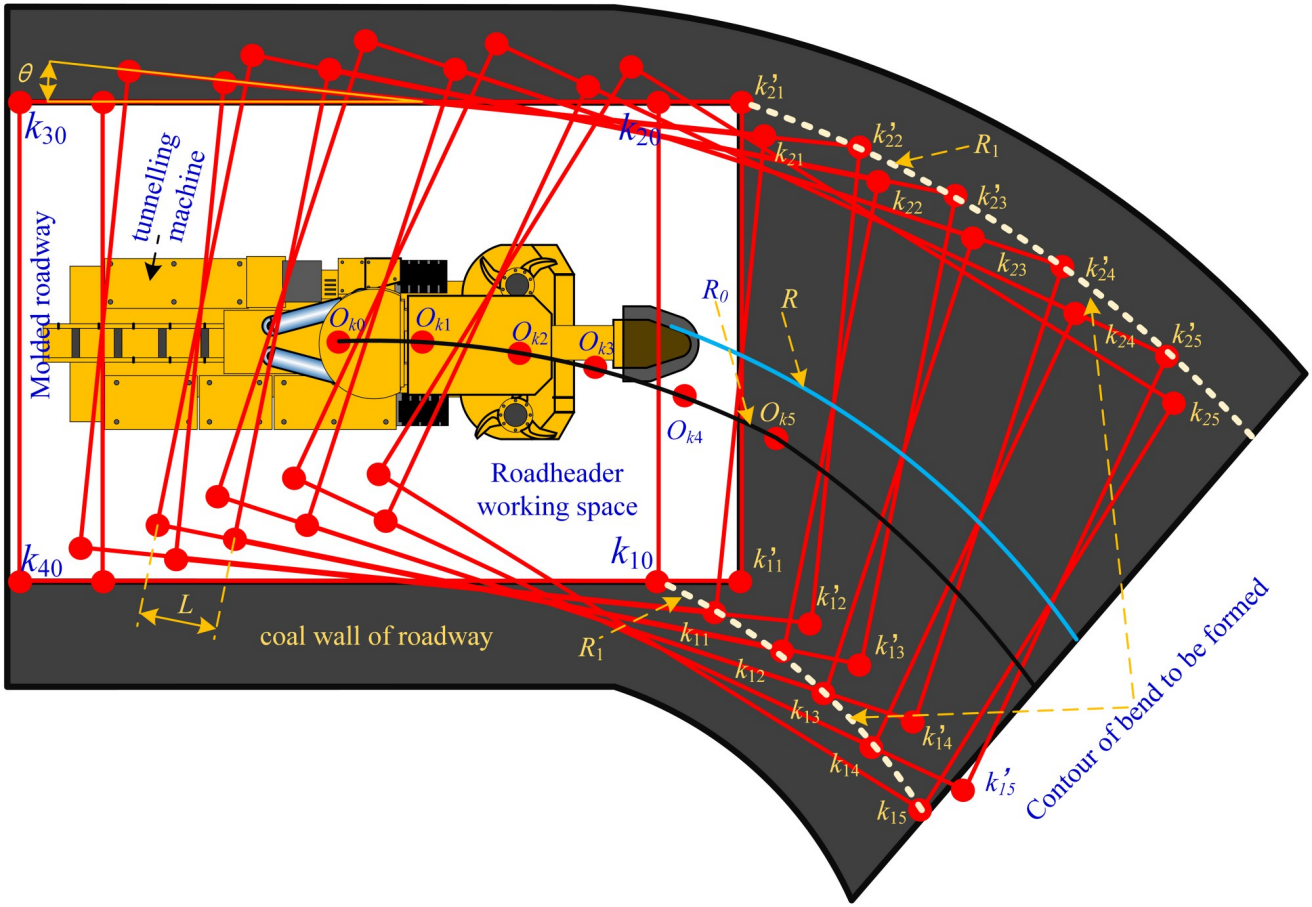
Roadway bending forming principle.

This topic takes roadway tunneling machinery as the carrier to study the tunneling process of roadway bend. The key technology of roadway forming is based on the accurate monitoring and control of the position and attitude of the roadheader [24]. Coal mine roadway forming is gradually formed by cutting multiple single sections by roadheader [25]. Therefore, the contour forming of the roadway curve can be understood as the trajectory of the working space of the roadheader under a certain feed law and rotation law. This paper only studies the problem of roadway curve forming when the roadheader is driving in the horizontal direction. Roadway bend forming can be mathematically expressed as five steps:

1. The initial working space *k*_10_ ~ *k*_40_ is formed by tunneling machine;
2. The work space The workspace rotates an angle *θ*;
3. After that, the roadheader digs forward, so that the working space of the roadheader advances to a given distance *L*;
4. Trimming the roadway contour to make the contour more flat and get the arc shape contour;
5. Repeat steps 1–4 for n times until the completion of the curve excavation. Curve forming diagram is shown in Fig 1.

In Fig 1: *k*_1_~*k*_4_ represents the working space of the roadheader; *O*_*k*_ is the rotation center of the workspace. Combined with the actual working conditions of roadway cutting, it can be seen that: The roadheader workspace *k*_1_*k*_2_*k*_3_*k*_4_ set in this paper is an extended meaning. When the roadheader forms the roadway, only the straight line *k*_1_*k*_2_ actually exists, so the maximum turning radius of the roadway is actually determined by the trajectory of the point *k*_2_, and the minimum turning radius of the roadway is actually determined by the trajectory of the point *k*_1_.

## 3 Curve mathematical model

### 3.1 Key point coordinates and curve radius calculation

**Fig 2:**
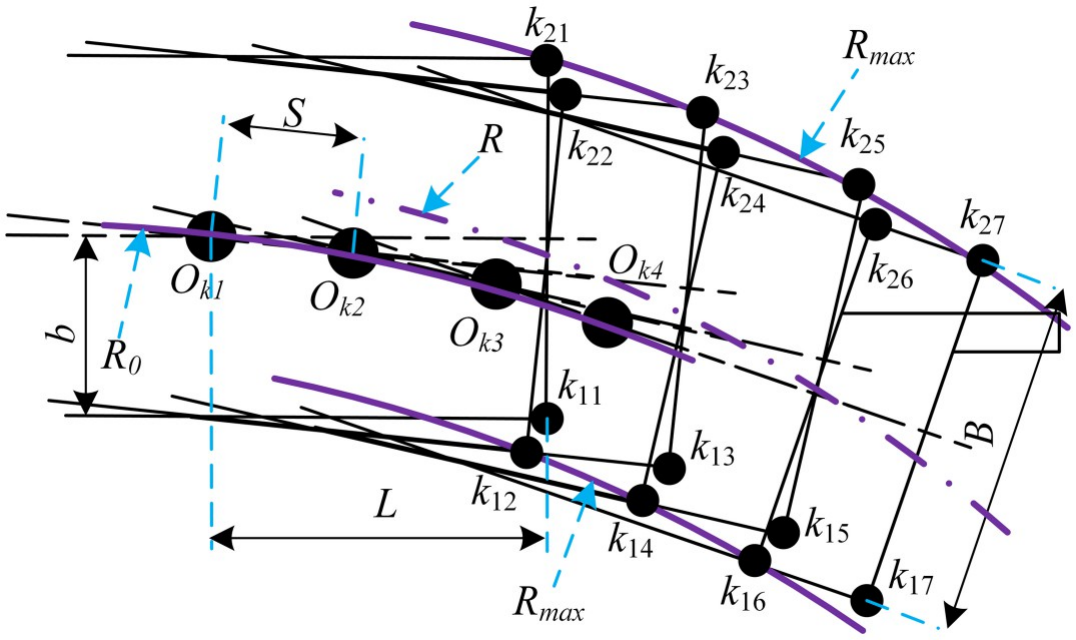
Curve simplified model.

According to the first section, the forming process of the bend is a cyclic process. Therefore, when solving the mathematical model of the bend, it can be simplified to the simplified model shown in Fig 2.

It can be seen from Fig 1 that the formation of the roadway curve is mainly affected by the points *k*_1_ and *k*_2_, the feed step size *S*, the rotation angle *θ* and the rotation center *O*_*k*_ of the roadheader working space. Based on the geometric relationship shown in Fig 2, the initial position is set as:

$$\left\{\begin{array}{l} O_{k1}=(x_{O_{{}_{k1}}},y_{O_{k1}})\\ k_{11}=(x_{O_{{}_{k1}}}+L,y_{O_{k1}}-b)\\ k_{21}=(x_{O_{{}_{k1}}}+L,y_{O_{k1}}+(B-b)) \end{array}\right.$$ (1)

Where: *k*_1_ and *k*_2_ workspace boundary points; *L* is the distance from the center of rotation to the boundary *k*_1_*k*_2_; *B* and *b* are the positions of the workspace rotation center in the *k*_1_*k*_2_ direction.

Combined with the roadway forming principle and the geometric relationship shown in Fig 2, the coordinate transformation formulas of points k1, k2 and Ok can be obtained as Eq (2).

$$\left\{\begin{array}{c} \vdots \\ k_{n(i)}=\left\{\begin{array}{l} x_{n(i)}=[x_{n(i-1)}-x_{o_{k_{\left(i-1\right)}}}]\cdot \text{cos}(-\theta )-\\ {}[y_{n(i-1)}-y_{o_{k_{\left(i-1\right)}}}]\cdot \text{sin}(-\theta )+x_{o_{k_{i-1}}}\\ y_{n(i)}=[y_{n(i-1)}-y_{o_{k_{\left(i-1\right)}}}]\cdot \text{cos}(-\theta )+\\ {}[x_{n(i-1)}-x_{o_{k_{\left(i-1\right)}}}]\cdot \text{sin}(-\theta )+y_{o_{k_{i-1}}} \end{array}\right.\\ O_{k(i)}=O_{k(i-1)}\\ k_{n(i+1)}=\left\{\begin{array}{l} x_{n(i)}+S\cdot \text{cos}[\frac{\theta (i-1)}{2}]\\ y_{n(i)}-S\cdot \text{sin}[\frac{\theta (i-1)}{2}] \end{array}\right.\\ O_{k(i+1)}=\left\{\begin{array}{l} x_{o_{k(i)}}+S\cdot \text{cos}[\frac{\theta (i-1)}{2}]\\ y_{o_{k(i)}}-S\cdot \text{sin}[\frac{\theta (i-1)}{2}] \end{array}\right.\\ \vdots \end{array}\right.$$ (2)

where *n* = 1, 2; *i* is a natural number greater than 1; *θ* is the rotation angle of working space; *S* is the feed rate of roadheader tunneling once.

In the ideal state, the trajectory of *k*_1_ and *k*_2_ can be obtained by solving Eqs (1) and (2). The maximum turning radius Rmax of the theoretical roadway can be obtained by bringing any three points in the point set {*k*_21_, *k*_23_, *k*_25_,…} into the circle. The minimum turning radius Rmin of the theoretical roadway can be obtained by bringing any three points in the point set {*k*_12_, *k*_14_, *k*_16_,…} into the circle.

Considering that in the actual working conditions, the control of each feed step and rotation angle of the roadheader cannot be consistent with the theoretical value, there will always be an unavoidable working condition error. In order to make the theoretical calculation more instructive for production, Eq (2) is rewritten as:

$$\left\{\begin{array}{c} \vdots \\ k_{n(i)}=\left\{\begin{array}{l} x_{n(i)}=[x_{n(i-1)}-x_{o_{k_{\left(i-1\right)}}}]\cdot \text{cos}[-(\theta +\varepsilon_{i})]-\\ {}[y_{n(i-1)}-y_{o_{k_{\left(i-1\right)}}}]\cdot \text{sin}[-(\theta +\varepsilon_{i})]+x_{o_{k_{i-1}}}\\ y_{n(i)}=[y_{n(i-1)}-y_{o_{k_{\left(i-1\right)}}}]\cdot \text{cos}[-(\theta +\varepsilon_{i})]+\\ {}[x_{n(i-1)}-x_{o_{k_{\left(i-1\right)}}}]\cdot \text{sin}[-(\theta +\varepsilon_{i})]+y_{o_{k_{i-1}}} \end{array}\right.\\ O_{k(i)}=O_{k(i-1)}\\ k_{n(i+1)}=\left\{\begin{array}{l} x_{n(i)}+(S+\varepsilon_{i})\cdot \text{cos}[\frac{\left(i-1)(\theta +\varepsilon_{i+1}\right)}{2}]\\ y_{n(i)}-(S+\varepsilon_{i})\cdot \text{sin}[\frac{\left(i-1)(\theta +\varepsilon_{i+1}\right)}{2}] \end{array}\right.\\ O_{k(i+1)}=\left\{\begin{array}{l} x_{o_{k(i)}}+(S+\varepsilon_{i})\cdot \text{cos}[\frac{\left(i-1)(\theta +\varepsilon_{i+1}\right)}{2}]\\ y_{o_{k(i)}}-(S+\varepsilon_{i})\cdot \text{sin}[\frac{\left(i-1)(\theta +\varepsilon_{i+1}\right)}{2}] \end{array}\right.\\ \vdots \end{array}\right.$$ (3)

In the formula, *ε*_*i*_ represents the error under each working condition.

The solution (3) can obtain the trajectory of *k*_1_ and *k*_2_ considering the working condition error. At this time, the least square method is used to fit the point set {*k*_21_, *k*_23_, *k*_25_,…} and the point set {*k*_12_, *k*_14_, *k*_16_,…}, and finally the roadway boundary curve is obtained. The fitting objective function is:

$$f=\sum_{i=1,j=1}^{m,n}{\left[{\left(x_{k_{j(i)}}-x_{j}\right)}^{2}+{\left(y_{k_{j(i)}}-y_{j}\right)}^{2}-R_{j}^{2}\right]}^{2}$$ (4)

In the formula: *m* is equal to the number of elements of the point set, *n* is equal to 2. The *x*_*kj*(*i*)_ and *y*_*kj*(*i*)_ are the horizontal and vertical coordinates of the trajectory points, respectively. *x*_*j*,_ *y*_*j*_ and *R*_*j*_ represent the coordinates and radius of the fitting circle respectively. The solution is as follows: first, the partial derivatives of the objective function are calculated for *x*_*j*,_ *y*_*j*_ and *R*_*j*_ respectively, and let them be zero, three equations are obtained, and the corresponding values can be obtained by solving the corresponding equations.

## 4 Simulation analysis of roadway curve

### 4.1 Simulation process

**Fig 3:**
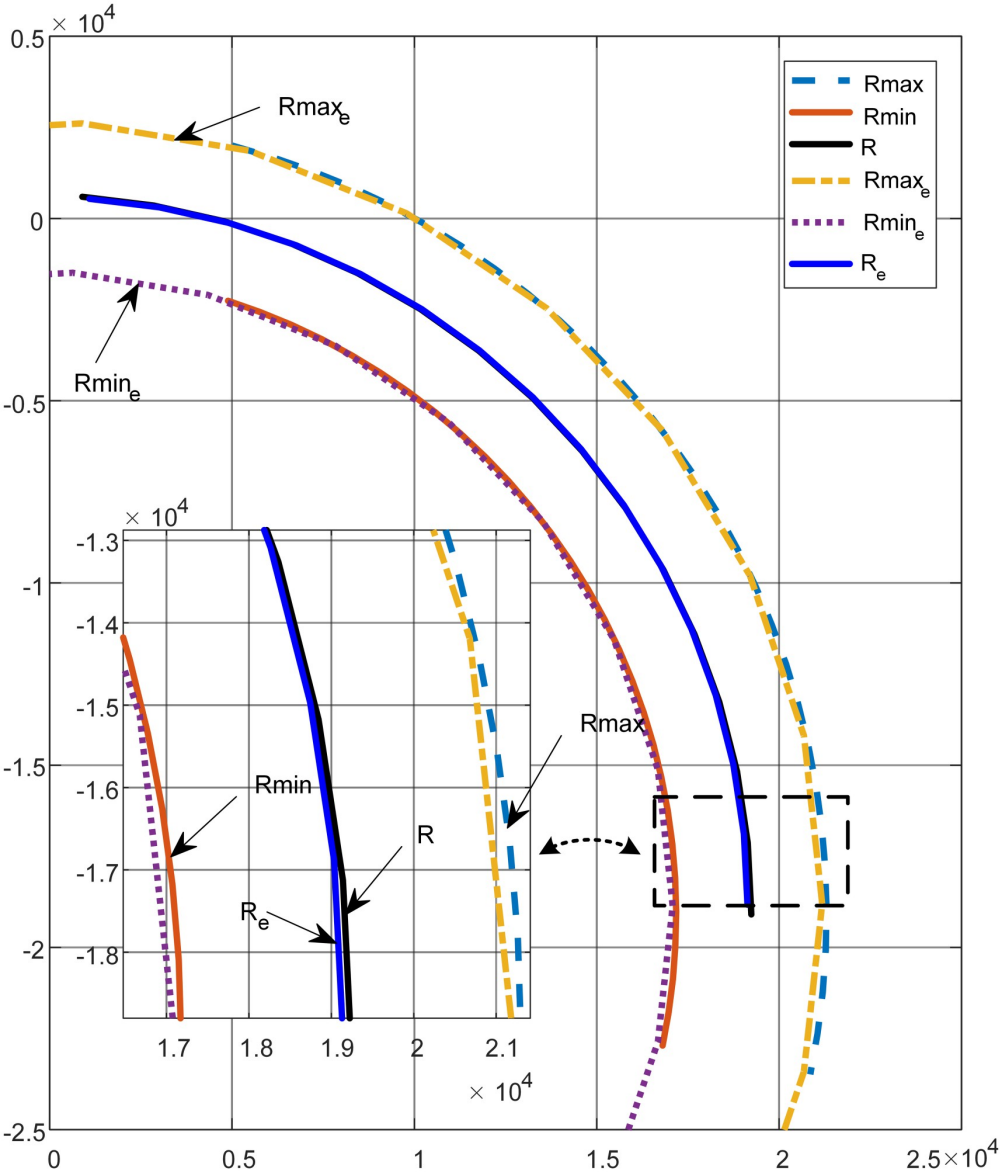
Roadway curve contour simulation.

Based on the research of cantilever roadheader, this paper discusses the influence of single feed and workspace angle of roadheader on the radius of roadway curve. During the excavation process, the single feed distance of the cantilever roadheader in the medium hard rock is 0.8 ~ 1.2 m. The common width of roadway is 4.5 ~ 6m. The determination of workspace angle mainly depends on the turning angle required by the roadway. Considering that this paper focuses on the influence of feed angle change on the turning radius, the single angle change interval is set to [1°, 5°]. The feed rate is 1m, the angle is 2°, the width of the roadway is 5m, and the distance between the center of the roadway space rotation is *L* = 3.5 ~ 7mm. The curve of the roadway is obtained by matlab solution as shown in Fig 3:

In Fig 3, R_max_, R_min_ and R are calculated values without introducing the influence of working condition error. R_max_e_, R_min_e_ and R__e_ are all calculated values under the influence of working condition error. Analysis of Fig 3 shows: The mathematical creation of the roadway curve deduced in the second section is correct, which can be used to analyze the influence of the basic parameters of the roadway and the change of the working space on the radius of the curve. The working condition error model can reasonably evaluate the actual working condition, and the least squares model is reliable. The simulation results are consistent with the expectations shown in Fig 2.

### 4.2 Evaluation method of roadway bend radius

In this paper, the mean value R_avg_ of the inner and outer contour radius of the roadway is used as the radius of the roadway, and the influence of various parameters on the radius of the curve is analyzed by R_avg_ evaluation index. The formula is as follows:

$$R_{avg}=\frac{1}{2}(R_{{\text{max}}_{e}}+R_{{\text{min}}_{e}})$$ (5)

In the formula: R_max_e_ and R_min_e_ are the outer contour radius of the roadway under the influence of error.

### 4.3 The influence of different parameters on the radius of the bend

**Fig 4:**
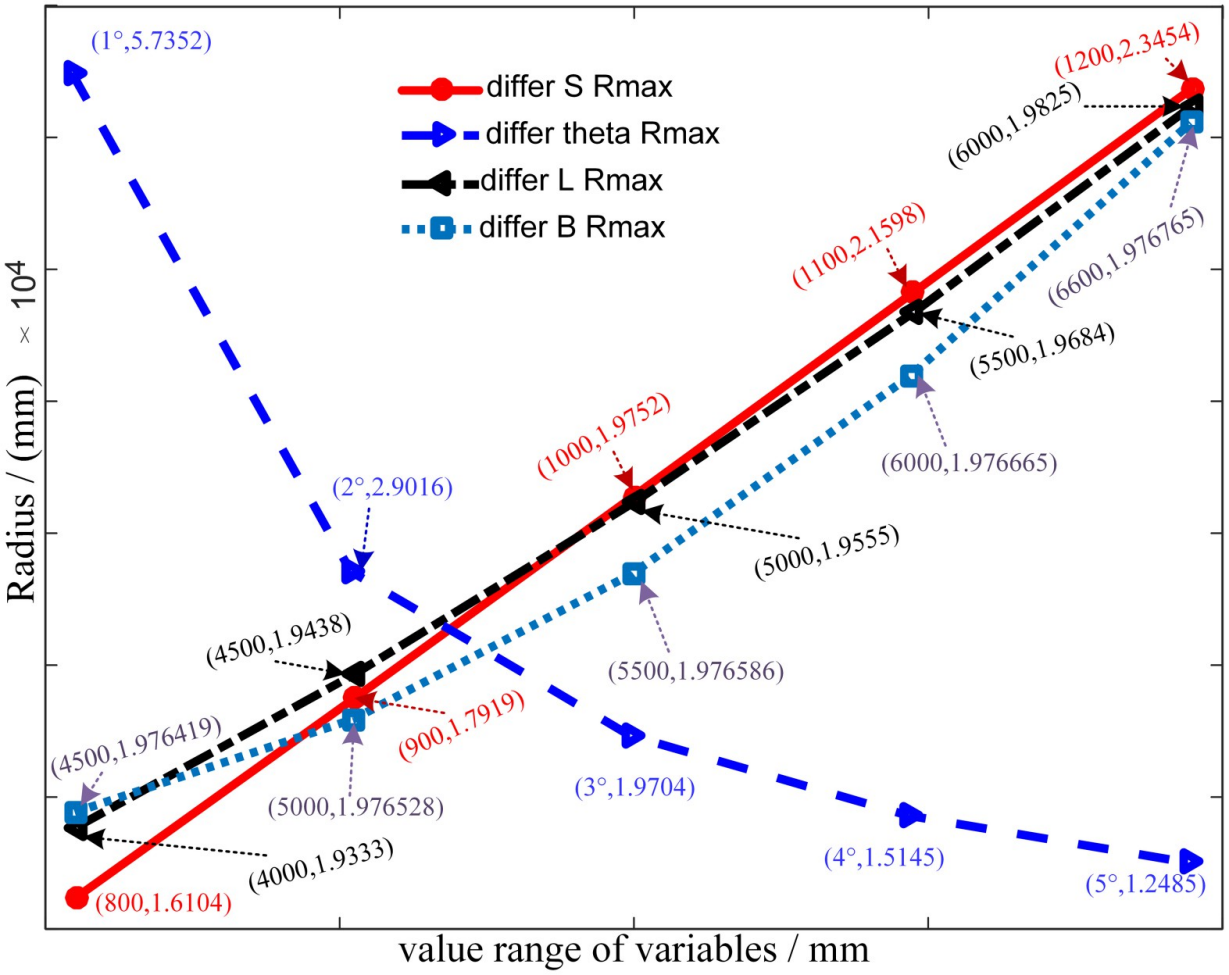
The influence of different variables on the radius of roadway curve.

According to the formula (2) and formula (3), the parameters that affect the radius of the roadway bend are mainly feed *S*, working space angle *θ*, working space position *L*, and roadway width *B* and *b*. Considering that the direct effect of *B* and *b* changes is only to change the position of the rotation center of the empty working space in the direction of *k*_1_*k*_2_, it is assumed that *b* = *B* / 2. In order to study the influence of different feed on the radius of the bend, the basic parameters are set as follows: working space angle *θ* = 3°, working space position *L* = 5m, roadway width *B* = 5m, feed *S* = 0.8m ~ 1.2m. When studying the influence of different feed rates on the radius of the bend, the basic parameters are set as follows: working space angle *θ* = 1° ~ 5°, working space position *L* = 5m, roadway width *B* = 5m, feed rate *S* = 1m. When studying the influence of different feed rates on the radius of the bend, the values of the basic parameters are set as follows: working space angle *θ* = 3°, working space position *L* = 5m, roadway width *B* = 4.5m ~ 6m, feed rate *S* = 0.8m ~ 1.2m. When studying the influence of different feed rates on the radius of the bend, the basic parameters are set as follows: working space angle *θ* = 3°, working space position *L* = 4m ~ 6m, roadway width *B* = 5m, feed rate *S* = 1m. Through theoretical calculation, the influence of only one variable change on the radius of the bend is shown in Fig 4.

According to the analysis of Fig 4, the radius of the roadway curve increases with the increase of the feed rate, the working space position of the roadheader and the required width of the roadway, and decreases with the increase of the working space angle. Among them, the linear relationship between the radius of the roadway curve and the feed rate is the strongest, followed by the working space position and the roadway width, and the linear relationship with the rotation angle is the worst. By analyzing the slope of the angle change curve, it can be seen that the greater the angle, the smaller the influence on the change of the radius of the roadway bend. By analyzing the change curve of roadway width, it can be seen that the change of roadway width has little influence on the radius of roadway bend. Compared with other variables, the influence of roadway width is the least obvious. The analysis of the change curve of the working space position shows that the influence of the change of the working space position on the radius of the roadway is slightly larger than that of the width of the roadway.

The results tell us: The radius of the roadway curve is mainly affected by the feed and the rotation angle. When we need to obtain a smaller roadway turning radius, we should select a larger corner, a smaller workspace position, a smaller roadway width, and a smaller feed rate.

## 5 RBF interpolation prediction

Considering that the calculation formula of the radius of the roadway bend deduced in this paper has an iterative solution process, it is not convenient and quick to use in practical engineering, which is not conducive to engineers to make a quick plan for roadway excavation. Therefore, in order to make the research more instructive, it is a feasible method to establish an interpolation model that can meet the needs to predict the radius of the roadway bend under multi-factor changes.

### 5.1 Establish interpolation model

The interpolation model is a mathematical model for approximating complex systems, which is usually used to replace the original model for calculation. The common methods of establishing interpolation models include polynomial response surface, polynomial interpolation, Kriging interpolation, neural network, support vector machine regression and radial basis function [26, 27].

The polynomial response surface modeling method uses multiple regression equations to fit the functional relationship between factors and response values, and seeks the optimal process parameters through the analysis of the regression equation. This method is suitable for the data in polynomial form.

The polynomial interpolation method is to use the function *f*(*x*) to insert the function value of several points in a certain interval to make an appropriate specific function, take the known value at these points, and use the value of this specific function at other points in the interval as the approximation of the function *f*(*x*). This method is suitable for data in the form of continuous function.

The Kriging interpolation method was first proposed in the field of geostatistics. It is a practical spatial estimation technique and also a kind of interpolation method. This method is suitable for data showing spatial correlation.

Neural network is a universal approximation model and the core of contemporary artificial intelligence and deep learning. This method uses the connection relationship between neurons to fit the nonlinear relationship between input and output, which is suitable for the case where the data presents a nonlinear form.

Support vector machine regression is a regression method based on statistical learning theory, which can deal with high-dimensional, nonlinear and sparse problems. This method uses kernel function to map the input to high-dimensional space, and then constructs a linear regression model in high-dimensional space to fit the relationship between input and output.

The radial basis function method uses the Euclidean distance between the test point and the unknown point as the input of the radial function. By using the Euclidean distance as the transfer mechanism, the multi-dimensional problem is transformed into a one-dimensional problem, thereby reducing the complexity of the model. This method is suitable for the case where the data presents spatial correlation and nonlinear form.

**Fig 5:**
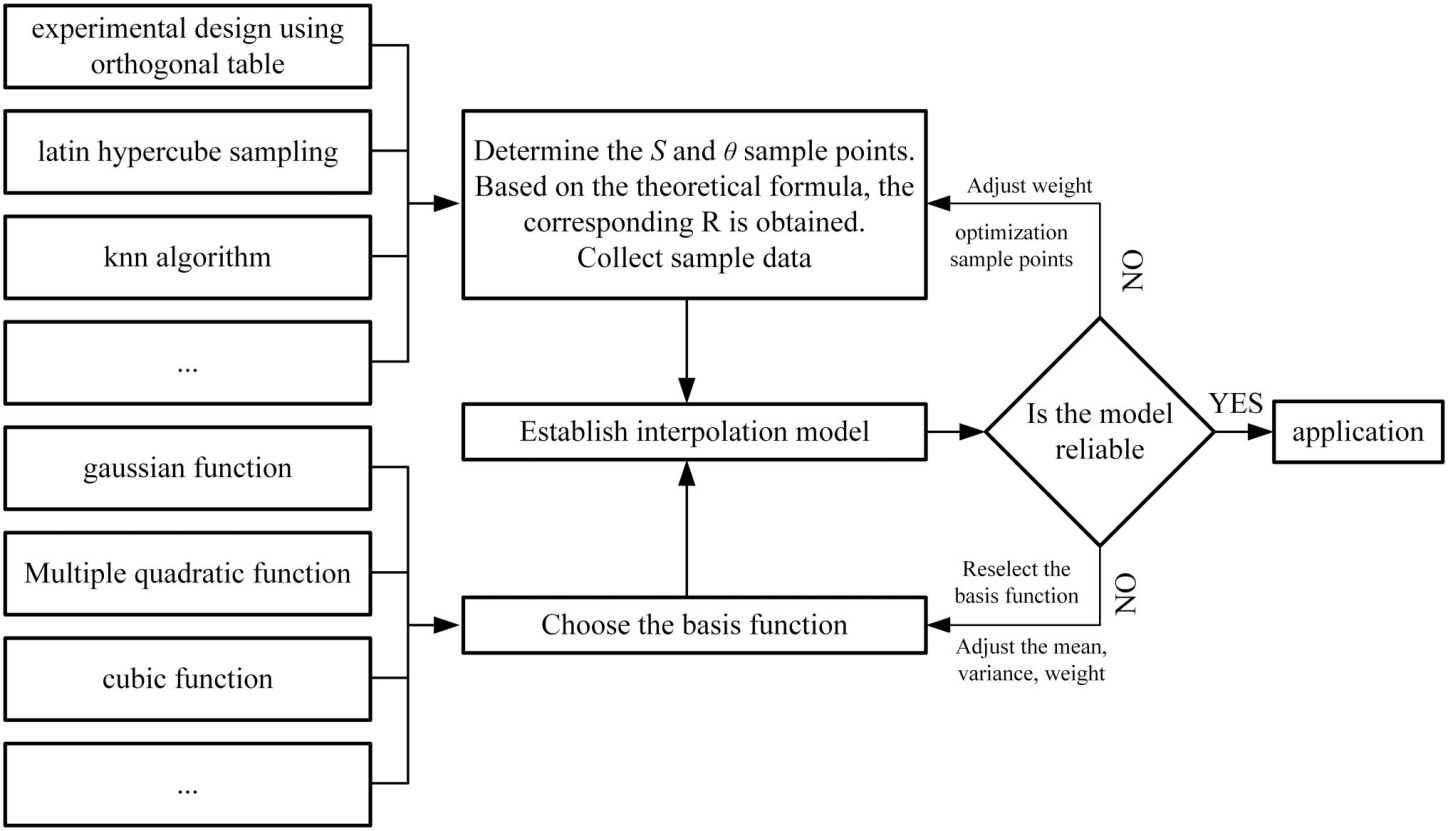
RBF interpolation model establishment process.

Generally, polynomial response surface, polynomial interpolation and Kriging interpolation are suitable for different forms of data, while neural network, support vector machine regression and radial basis function are suitable for nonlinear data. The analysis of the previous section shows that the main influencing factors of the radius of the roadway bend are the feed rate and the working space angle. In addition, in the actual working conditions, the width of the roadway and the position of the working space are rarely changed in the special sections. Therefore, when establishing the interpolation model, only the feed rate and the space angle are used as variables. The spatial rotation angle in the feed is defined as the X and Y axes in the space, and the radius value corresponding to each group variable is defined as the Z axis. At this time, a point cloud map can be obtained. This idea is used as the basis for establishing the interpolation model, which is suitable for the radial basis function method to establish the interpolation model. The idea and main process of interpolation model establishment are shown in Fig 5.

Based on the agent model establishment process shown in Fig 5. After several rounds of selection and verification, it was finally determined that the KNN algorithm (K Nearest Neighbors, classification method based on distance variables) was used to sample the sample points in different regions [28, 29]. That is, a certain amount of sample points are selected around each data point that needs to be interpolated, and the selected points are used to interpolate the points with the Gaussian basis function to establish the RBF interpolation model. The general formula of Gaussian basis function is as follows:

$$\begin{array}{l} p(x_{1},x_{2})=\prod_{i=1}^{2}p(x_{i})\\ =\frac{1}{2\pi \sigma_{1}\sigma_{2}}\text{exp}\left[-\frac{1}{2}\left(\frac{{\left(x_{1}-\mu_{1}\right)}^{2}}{\sigma_{1}}+\frac{{\left(x_{2}-\mu_{2}\right)}^{2}}{\sigma_{2}}\right)\right] \end{array}$$ (6)

In the formula: *x*_1_ and *x*_2_ represent the feed *S* and the space angle *θ* respectively; *μ*_1_, *μ*_1_ and *σ*_1_, *σ*_2_ are the mean and variance of the corresponding independent variables.

The Gaussian basis function has the following advantages: The output value of the Gaussian basis function is related to the distance from the center point. The closer to the center point, the larger the function value, the farther from the center point, the smaller the function value. This is conducive to more accurate prediction of sample points closer to the center point. Secondly, the Gaussian basis function has a good local approximation property, that is, only the points close to the query point have a real mapping effect on the input, and the point output far from the query point tends to 0. This can avoid the influence of sample points away from the query point on the prediction results. Combined with the KNN algorithm, the interpolation of sample points can be complementary to the Gaussian radial basis function.

### 5.2 Prediction of roadway bend radius

The above RBF interpolation model between the radius of roadway curve and multi-scale is established by combining KNN algorithm with radial basis function. The most intuitive way to test the prediction accuracy of RBF interpolation model is to take a set of independent variables and directly compare the difference between the theoretical calculation value and the calculated value of RBF interpolation model. And the results are displayed in the form of images.

**Fig 6:**
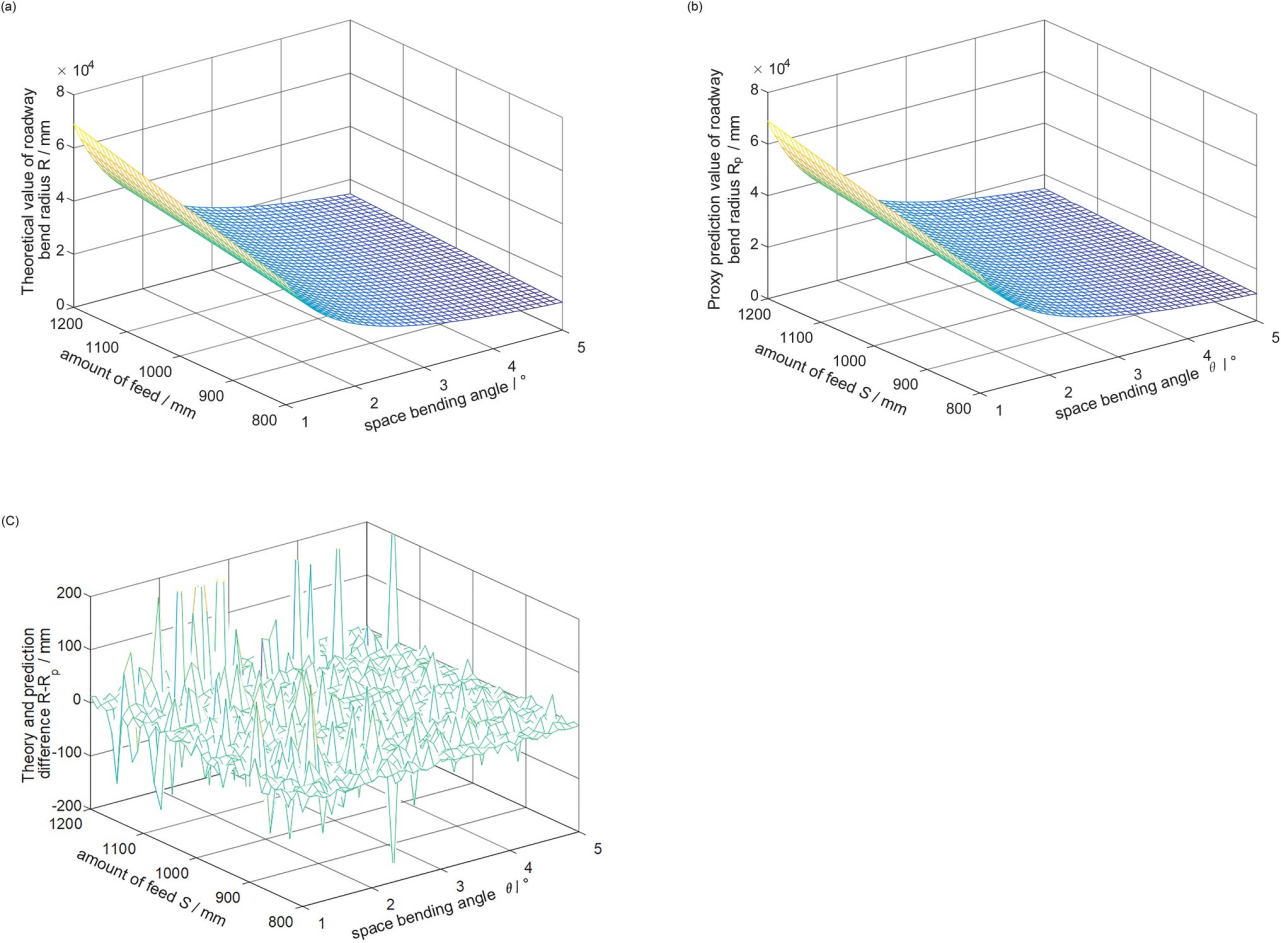
Theoretical calculation data and RBF interpolation model calculation data. (a) Theoretical value. (b) Interpolation calculated value. (c) The difference between the theoretical value and the predicted value.

The width of roadway *B* = 5m, the position of working space *L* = 5m, the change range of feed *S* is 0.8m ~ 1.2m, and the change of spatial angle *θ* is 1 ~ 5° (41 nodes are evenly taken in the value range of both groups). The data image obtained by theoretical calculation and RBF interpolation model is shown in Fig 6.

Comparing Fig 6(a) and 6(b), it can be seen that under the current parameter setting, the radius of the roadway curve is between 10 and 70 m. The radius of the roadway curve calculated by the RBF interpolation model is very close to the contour of the radius of the roadway curve obtained by theoretical calculation. Comparing the change trend of a variable alone, it is found that the change trend of the two is consistent. The roadway radius increases with the increase of feed rate and the decrease of rotation angle. It can be seen that RBF interpolation model calculation instead of theoretical calculation is feasible in trend prediction.

It can be seen from Fig 6(c) that the calculated value of RBF interpolation model is similar to the theoretical value, and the difference between the two is mainly concentrated in the range of 0–200 mm. And the larger error is mainly distributed at the feed rate greater than 1.1 m. Compared with the radius of the roadway curve, the difference between the two can be ignored. It can be seen that the radius of the roadway bend calculated by the RBF interpolation model is not much different from the theoretically calculated radius of the roadway bend, and the error distribution is normal. The calculated value of RBF interpolation model can replace the theoretical calculation numerically. It can be seen from the above analysis that the RBF interpolation model has reliable accuracy and can replace the theoretical calculation to simplify the calculation. Applied to the actual project, it is helpful for engineers to make a quick choice of roadway excavation.

## 6 Conclusion

This paper mainly analyzes the tunneling process of roadway curve, and based on the analysis of this process, a mathematical model of roadway curve considering working condition error is established. On the basis of the established mathematical model, the influence of the position of roadway excavation working space, the required width of roadway, the amount of excavation feed and the change of the rotation angle of roadway excavation working space on the formation of roadway bend is studied with the radius of roadway bend as the evaluation index. Considering that the derived formula for calculating the radius of roadway bend is complex, and there is no need for too many repetitive and meaningless calculations for practical operations, in order to make the research more instructive for practical projects, this paper establishes a corresponding RBF interpolation model based on KNN algorithm and radial basis function interpolation method to simplify the calculation process. Research shows that:

1. The radius of roadway curve increases with the increase of feed rate, working space position of roadheader and required width of roadway, and decreases with the increase of working space angle. When we need to obtain a smaller roadway turning radius, we should select a larger corner, a smaller workspace position, a smaller roadway width, and a smaller feed rate.
2. Compared with the working space of roadway excavation and the required width of roadway, the radius of roadway bend is mainly affected by feed and rotation angle. It is not obvious to change the turning radius of roadway by changing the working space and width of roadway.
3. The value calculated by RBF interpolation model is similar to the theoretical value, the change trend is consistent, and the error is within a reasonable range. Engineers can directly use the RBF interpolation model to design and calculate the radius of the roadway curve. Compared with theoretical calculation, the calculation of RBF interpolation model can simplify the calculation, which is helpful for engineers to make a quick choice of roadway excavation.

## Supporting information

S1 Text (TXT)

S2 Text (TXT)

S1 File (DOCX)

S1 Data (CSV)
