# Supplementary material for: Curve forming prediction of coal mine roadway based on RBF interpolation
Source: PLoS One. 2023 Jul 21;18(7):e0288753. doi: 10.1371/journal.pone.0288753 (PMC10361495; doi:10.1371/journal.pone.0288753)
Supplement: S1 File — (DOCX) [file pone.0288753.s003.docx]

Table 1 The trajectory of point O_k_, k_2i_ and point k_1i_ under different parameters

a）differ B

| k_1i_ | | k_2i_ | | O_i_ | |
| --- | --- | --- | --- | --- | --- |
| X | Y | X | X | Y | X |
| 5000 | -3250 | 5000 | 3250 | 0 | 0 |
| 4823.72237382873 | -3506.30895105153 | 5162.67227223416 | 2984.84756888934 | 0 | 0 |
| 5799.80410223602 | -3557.27718786152 | 6138.75400064144 | 2933.87933207936 | 976.081728407286 | -50.9682368099873 |
| 5609.32529523123 | -3805.37285678431 | 6288.15495568844 | 2659.08305938401 | 976.081728407286 | -50.9682368099873 |
| 6606.99713765635 | -3910.42694169800 | 7285.82679811357 | 2554.02897447031 | 1973.75357083241 | -156.022321723680 |
| 6403.08108326020 | -4148.96081206513 | 7421.03418655890 | 2270.83447123515 | 1973.75357083241 | -156.022321723680 |
| 7386.36062830725 | -4305.51206900720 | 8404.31373160594 | 2114.28321429308 | 2957.03311587945 | -312.573578665751 |
| 7172.41440380216 | -4530.75572236594 | 8523.30340100115 | 1827.31780011291 | 2957.03311587945 | -312.573578665751 |
| 8185.78771031620 | -4745.05218268464 | 9536.67670751520 | 1613.02133979422 | 3970.40642239350 | -526.870038984449 |
| 7960.06936591724 | -4959.14818877080 | 9640.69952932301 | 1319.82324402380 | 3970.40642239350 | -526.870038984449 |
| 8913.19241126794 | -5213.59060090643 | 10593.8225746737 | 1065.38083188818 | 4923.52946774420 | -781.312451120073 |
| 8676.25517798406 | -5415.91596638486 | 10682.5338034263 | 766.708563464389 | 4923.52946774420 | -781.312451120073 |
| 9634.41115433990 | -5726.55696593493 | 11640.6897797821 | 456.067563914326 | 5881.68544410005 | -1091.95345067014 |
| 9386.87379355709 | -5916.49017815295 | 11713.7726269043 | 152.738917816918 | 5881.68544410005 | -1091.95345067014 |
| 10327.2117424803 | -6277.19848916507 | 12654.1105758276 | -207.969393195196 | 6822.02339302329 | -1452.66176168225 |
| 10070.5788717229 | -6453.60040375845 | 12711.1348331977 | -514.116906490368 | 6822.02339302329 | -1452.66176168225 |
| 10983.4235213641 | -6858.85748246156 | 13623.9794828389 | -919.373985193476 | 7734.86804266451 | -1857.91884038536 |
| 10718.5244866581 | -7021.27958937583 | 13664.8719583720 | -1227.40035557011 | 7734.86804266451 | -1857.91884038536 |
| 11623.1119704050 | -7479.64703135215 | 14569.4594421189 | -1685.76779754643 | 8639.45552641135 | -2316.28628236168 |
| 11348.9580108103 | -7628.63946028914 | 14594.3214779376 | -1996.79997693777 | 8639.45552641135 | -2316.28628236168 |
| 12220.9545370056 | -8131.72701908540 | 15466.3180041329 | -2499.88753573403 | 9511.45205260662 | -2819.37384115794 |
| 11937.8648195289 | -8266.85225639893 | 15474.8793374649 | -2813.45622274156 | 9511.45205260662 | -2819.37384115794 |
| 12781.0528026005 | -8817.69728120948 | 16318.0673205365 | -3364.30124755210 | 10354.6400356782 | -3370.21886596849 |
| 12492.4362968089 | -8937.29414351733 | 16310.1937802241 | -3676.61663982754 | 10354.6400356782 | -3370.21886596849 |
| 13305.1637483743 | -9528.28980051513 | 17122.9212317894 | -4267.61229682534 | 11167.3674872435 | -3961.21452296629 |
| 13011.6720341187 | -9632.28503375838 | 17098.8103975072 | -4578.04918661814 | 11167.3674872435 | -3961.21452296629 |
| 13795.6226820070 | -10264.7499897384 | 17882.7610453956 | -5210.51414259818 | 11951.3181351319 | -4593.67947894632 |
| 13495.4547159739 | -10353.7265464368 | 17842.2144888569 | -5520.95513896822 | 11951.3181351319 | -4593.67947894632 |
| 14239.4125043737 | -11026.3429857729 | 18586.1722772567 | -6193.57157830428 | 12695.2759235317 | -5266.29591828238 |
| 13935.6191848871 | -11099.3095985347 | 18529.5226846991 | -6500.82608389476 | 12695.2759235317 | -5266.29591828238 |
| 14648.6049625009 | -11813.1009673781 | 19242.5084623129 | -7214.61745273824 | 13408.2617011455 | -5980.08728712586 |
| 14342.8088287550 | -11869.8344520114 | 19170.2105802637 | -7517.11204763830 | 13408.2617011455 | -5980.08728712586 |
| 15007.1407178258 | -12602.9101509715 | 19834.5424693344 | -8250.18774659848 | 14072.5935902162 | -6713.16298608605 |
| 14696.8668060881 | -12643.8276159522 | 19745.9727121789 | -8550.35359635967 | 14072.5935902162 | -6713.16298608605 |
| 15306.0404482961 | -13399.3993672252 | 20355.1463543870 | -9305.92534763269 | 14681.7672324242 | -7468.73473735907 |
| 14995.4910497007 | -13423.9070578275 | 20251.4681259822 | -9599.68099578512 | 14681.7672324242 | -7468.73473735907 |
| 15586.8869110074 | -14234.3176456824 | 20842.8639872888 | -10410.0915836400 | 15273.1630937309 | -8279.14532521391 |
| 15274.8731868613 | -14242.5753009562 | 20723.7406313392 | -10698.5883409760 | 15273.1630937309 | -8279.14532521391 |
| 15827.2079541053 | -15092.5870935240 | 21276.0753985831 | -11548.6001335438 | 15825.4978609748 | -9129.15711778172 |
| 15515.5600643436 | -15084.5276824250 | 21142.1886563925 | -11830.1382332138 | 15825.4978609748 | -9129.15711778172 |
| 16016.3739026903 | -15948.9012681797 | 21643.0024947393 | -12694.5118189685 | 16326.3116993216 | -9993.53070353645 |
| 15704.2487380795 | -15924.4275726660 | 21493.9212386896 | -12969.8222968841 | 16326.3116993216 | -9993.53070353645 |
| 16156.9330672987 | -16819.6768240797 | 21946.6055679089 | -13865.0715482978 | 16778.9960285408 | -10888.7799549502 |
| 15847.2835250476 | -16778.9762004784 | 21783.6998235632 | -14131.5318609844 | 16778.9960285408 | -10888.7799549502 |
| 16250.1542895092 | -17684.7754886890 | 22186.5705880248 | -15037.3311491950 | 17181.8667930024 | -11794.5792431608 |
| 15942.8530992012 | -17627.8754868444 | 22009.8138640875 | -15295.0687916098 | 17181.8667930024 | -11794.5792431608 |
| 16299.4943011259 | -18560.3518625315 | 22366.4550660122 | -16227.5451672970 | 17538.5079949271 | -12727.0556188479 |
| 15995.4085890703 | -18487.3806290921 | 22176.3165056021 | -16475.8197109603 | 17538.5079949271 | -12727.0556188479 |
| 16305.8300518267 | -19449.4738427794 | 22486.7379683584 | -17437.9129246476 | 17848.9294576835 | -13689.1488325353 |
| 16004.7781941853 | -19360.2691907483 | 22282.9956165634 | -17676.8245087501 | 17848.9294576835 | -13689.1488325353 |
| 16254.2021606801 | -20319.7425974240 | 22532.4195830582 | -18636.2979154258 | 18098.3534241782 | -14648.6222392110 |
| 15962.0037644812 | -20216.2528062936 | 22319.2165586122 | -18861.3190477146 | 18098.3534241782 | -14648.6222392110 |
| 16176.7576120805 | -21180.4861038355 | 22533.9704062115 | -19825.5523452565 | 18313.1072717776 | -15612.8555367529 |
| 15887.0101342928 | -21060.4745285225 | 22306.6634000046 | -20041.6261845399 | 18313.1072717776 | -15612.8555367529 |
| 16037.1263869064 | -22049.0736023793 | 22456.7796526182 | -21030.2252583967 | 18463.2235243911 | -16601.4546106097 |
| 15754.4538410373 | -21914.1814812989 | 22218.7458019020 | -21233.7922950969 | 18463.2235243911 | -16601.4546106097 |
| 15853.5422232309 | -22901.5250956506 | 22317.8341840955 | -22221.1359094485 | 18562.3119065847 | -17588.7982249614 |
| 15577.9131535009 | -22751.7299582494 | 22069.0401831179 | -22412.2157680073 | 18562.3119065847 | -17588.7982249614 |
| 15622.8843034057 | -23748.4601105288 | 22114.0113330227 | -23408.9459202867 | 18607.2830564896 | -18585.5283772409 |

b）differ L

| k_1i_ | | k_2i_ | | O_i_ | |
| --- | --- | --- | --- | --- | --- |
| X | Y | X | X | Y | X |
| 6000 | -2000 | 6000 | 2000 | 0 | 0 |
| 5887.07305072525 | -2311.35693812629 | 6096.47260345597 | 1683.15827993411 | 0 | 0 |
| 6889.20026166816 | -2363.89021896147 | 7098.59981439888 | 1630.62499909894 | 1002.12721094291 | -52.5332808351723 |
| 6759.97724343084 | -2669.24171002225 | 7178.43229444550 | 1308.80999530792 | 1002.12721094291 | -52.5332808351723 |
| 7756.29861369311 | -2774.10399775322 | 8174.75366470777 | 1203.94770757696 | 1998.44858120518 | -157.395568566137 |
| 7611.02337670273 | -3072.70072668350 | 8237.69151247363 | 877.905182238502 | 1998.44858120518 | -157.395568566137 |
| 8597.80643466139 | -3229.44613051331 | 9224.47457043229 | 721.159778408693 | 2985.23163916384 | -314.140972395946 |
| 8437.28792017289 | -3519.61789614845 | 9270.16281822258 | 392.711257207483 | 2985.23163916384 | -314.140972395946 |
| 9416.33216792666 | -3728.04122416508 | 10249.2070659764 | 184.287929190851 | 3964.27588691761 | -522.564300412578 |
| 9240.59589952338 | -4009.74756553873 | 10277.6418560779 | -146.518904950037 | 3964.27588691761 | -522.564300412578 |
| 10199.2447394112 | -4267.35758078721 | 11236.2906959658 | -404.128920198519 | 4922.92472680548 | -780.174315661061 |
| 10008.9195872106 | -4539.51684859588 | 11247.3261348905 | -736.051425765848 | 4922.92472680548 | -780.174315661061 |
| 10961.8942001105 | -4850.14802207035 | 12200.3007477904 | -1046.68259924032 | 5875.89933970542 | -1090.80548913553 |
| 10758.2785223604 | -5111.05094392775 | 12193.9499587047 | -1377.57434337633 | 5875.89933970542 | -1090.80548913553 |
| 11688.4426731766 | -5467.91559101072 | 13124.1141095209 | -1734.43899045930 | 6806.06349052164 | -1447.67013621850 |
| 11472.9305939015 | -5716.20049682162 | 13100.6753895874 | -2062.37416267306 | 6806.06349052164 | -1447.67013621850 |
| 12406.1897435819 | -6128.40276482894 | 14033.9345392678 | -2474.57643068038 | 7739.32264020201 | -1859.87240422582 |
| 12177.3956954636 | -6365.81376604592 | 13993.3459015033 | -2801.78166052495 | 7739.32264020201 | -1859.87240422582 |
| 13089.1912069243 | -6828.10685672923 | 14905.1414129640 | -3264.07475120826 | 8651.11815166268 | -2322.16549490913 |
| 12846.7504634405 | -7054.67746048996 | 14847.1311804335 | -3590.79568030709 | 8651.11815166268 | -2322.16549490913 |
| 13720.8643469345 | -7560.66772446664 | 15721.2450639274 | -4096.78594428376 | 9525.23203515664 | -2828.15575888581 |
| 13468.3098481350 | -7773.06600688803 | 15646.6404343407 | -4418.23726950054 | 9525.23203515664 | -2828.15575888581 |
| 14303.0853820690 | -8312.86590568380 | 16481.4159682746 | -4958.03716829631 | 10360.0075690906 | -3367.95565768158 |
| 14038.7618910644 | -8512.54180610584 | 16389.7627157242 | -5276.37198323669 | 10360.0075690906 | -3367.95565768158 |
| 14851.0292956118 | -9103.04485113595 | 17202.0301202716 | -5866.87502826680 | 11172.2749736379 | -3958.45870271169 |
| 14576.4600769955 | -9288.70483043212 | 17093.7707329562 | -6180.14454287351 | 11172.2749736379 | -3958.45870271169 |
| 15356.8381484754 | -9921.52786728792 | 17874.1488044361 | -6812.96757972931 | 11952.6530451179 | -4591.28173956749 |
| 15074.1021398457 | -10091.8776662851 | 17750.1698769307 | -7118.88902410492 | 11952.6530451179 | -4591.28173956749 |
| 15819.2584205155 | -10759.7596591994 | 18495.3261576005 | -7786.77101701918 | 12697.8093257876 | -5259.16373248175 |
| 15529.2063109414 | -10914.5320770250 | 18356.1479206366 | -8084.62021702413 | 12697.8093257876 | -5259.16373248175 |
| 16242.5745282284 | -11619.9806454767 | 19069.5161379236 | -8790.06878547583 | 13411.1775430746 | -5964.61230093345 |
| 15943.3514335221 | -11760.1360381410 | 18914.2308977572 | -9081.72695643095 | 13411.1775430746 | -5964.61230093345 |
| 16620.5316224754 | -12509.5689924610 | 19591.4110867104 | -9831.15991075099 | 14088.3577320279 | -6714.04525525350 |
| 16314.0094764258 | -12634.0515316571 | 19420.8828933884 | -10114.6592297376 | 14088.3577320279 | -6714.04525525350 |
| 16945.5344557691 | -13412.7195812142 | 20052.4078727317 | -10893.3272792947 | 14719.8827113711 | -7492.71330481057 |
| 16632.6770332923 | -13521.0810674828 | 19867.1388265114 | -11167.7309217956 | 14719.8827113711 | -7492.71330481057 |
| 17212.7453174006 | -14319.3665601691 | 20447.2071106197 | -11966.0164144819 | 15299.9509954794 | -8290.99879749682 |
| 16892.2971355134 | -14411.8184950797 | 20246.3197260564 | -12232.2468715428 | 15299.9509954794 | -8290.99879749682 |
| 17430.8655883198 | -15254.4008463211 | 20784.8881788628 | -13074.8292227841 | 15838.5194482858 | -9133.58114873820 |
| 17111.0443078279 | -15328.7954696352 | 20573.6679284577 | -13326.2376897306 | 15838.5194482858 | -9133.58114873820 |
| 17623.1975415276 | -16198.2807344980 | 21085.8211621574 | -14195.7229545933 | 16350.6726819855 | -10003.0664136010 |
| 17298.7470392891 | -16256.1580499856 | 20860.9871496592 | -14436.6951083265 | 16350.6726819855 | -10003.0664136010 |
| 17758.9033192839 | -17147.9385642070 | 21321.1434296540 | -15328.4756225479 | 16810.8289619803 | -10894.8469278224 |
| 17430.1618822229 | -17189.0050712046 | 21082.7901842036 | -15558.5736903826 | 16810.8289619803 | -10894.8469278224 |
| 17840.0981854831 | -18111.3035409443 | 21492.7264874639 | -16480.8721601223 | 17220.7652652405 | -11817.1453975621 |
| 17510.1397117453 | -18135.0772134106 | 21243.0239119534 | -16697.8661823380 | 17220.7652652405 | -11817.1453975621 |
| 17867.0496295411 | -19061.8219184734 | 21599.9338297492 | -17624.6108874009 | 17577.6751830363 | -12743.8901026250 |
| 17536.5289166547 | -19068.3115769389 | 21339.4110545193 | -17828.1150419051 | 17577.6751830363 | -12743.8901026250 |
| 17851.1678585740 | -20030.0737535322 | 21654.0499964387 | -18789.8772184984 | 17892.3141249556 | -13705.6522792183 |
| 17519.4971138062 | -20019.2097135177 | 21382.1949766157 | -18980.1884333475 | 17892.3141249556 | -13705.6522792183 |
| 17775.4508282761 | -20985.6457006141 | 21638.1486910856 | -19946.6244204439 | 18148.2678394255 | -14672.0882663147 |
| 17444.5692914190 | -20957.3733317301 | 21356.4800402695 | -20122.5354474509 | 18148.2678394255 | -14672.0882663147 |
| 17650.6508658618 | -21946.3346487612 | 21562.5616147124 | -21111.4967644820 | 18354.3494138684 | -15661.0495833458 |
| 17323.5228464217 | -21901.0332842737 | 21273.6783804678 | -21271.5324845562 | 18354.3494138684 | -15661.0495833458 |
| 17483.6078032065 | -22888.1482970336 | 21433.7633372526 | -22258.6474973160 | 18514.4343706532 | -16648.1645961057 |
| 17156.4411858043 | -22825.2071335911 | 21134.2653452653 | -22404.5945370033 | 18514.4343706532 | -16648.1645961057 |
| 17249.5582363832 | -23795.8881817479 | 21227.3823958442 | -23375.2755851602 | 18607.5514212321 | -17618.8456442625 |
| 16928.0278941562 | -23716.3205906644 | 20922.4175674125 | -23504.5397176295 | 18607.5514212321 | -17618.8456442625 |
| 16978.7054320480 | -24693.0824684226 | 20973.0951053043 | -24481.3015953878 | 18658.2289591238 | -18595.6075220207 |

c）differ S

| k_1i_ | | k_2i_ | | O_i_ | |
| --- | --- | --- | --- | --- | --- |
| X | Y | X | X | Y | X |
| 5000 | -2000 | 5000 | 2000 | 0 | 0 |
| 4888.67011684770 | -2258.51820640001 | 5097.67022695868 | 1736.01793112255 | 0 | 0 |
| 6101.80034095499 | -2321.99099565224 | 6310.80045106597 | 1672.54514187032 | 1213.13022410729 | -63.4727892522298 |
| 5976.87878611279 | -2574.78711406116 | 6394.68216542777 | 1403.33308726923 | 1213.13022410729 | -63.4727892522298 |
| 7178.10673984690 | -2701.06069341575 | 7595.91011916189 | 1277.05950791464 | 2414.35817784140 | -189.746368606821 |
| 7039.85193828428 | -2947.42790789014 | 7665.70299697819 | 1003.30752394172 | 2414.35817784140 | -189.746368606821 |
| 8222.70810658381 | -3135.16206343086 | 8848.55916527772 | 815.573368401010 | 3597.21434614093 | -377.480524147534 |
| 8071.09435940251 | -3374.87900064988 | 8904.09192574576 | 537.424036496685 | 3597.21434614093 | -377.480524147534 |
| 9241.77600690387 | -3625.26504439168 | 10074.7735732471 | 287.037992754880 | 4767.89599364229 | -627.866567889339 |
| 9077.75632048597 | -3856.65952549733 | 10115.5830976381 | 6.35944683844161 | 4767.89599364229 | -627.866567889339 |
| 10222.1895345658 | -4165.24184662374 | 11260.0163117179 | -302.222874287973 | 5912.32920772211 | -936.448889015753 |
| 10046.9839886309 | -4386.75754806468 | 11285.9127794484 | -583.462207010629 | 5912.32920772211 | -936.448889015753 |
| 11180.2280933116 | -4755.66184080031 | 12419.1568841291 | -952.366499746257 | 7045.57331240278 | -1305.35318175138 |
| 10993.6028961546 | -4967.73797936896 | 12430.2739058904 | -1234.64590847320 | 7045.57331240278 | -1305.35318175138 |
| 12116.7217327225 | -5399.80800039612 | 13553.3927424582 | -1666.71592950036 | 8168.69214897064 | -1737.42320277854 |
| 11921.1106503723 | -5599.98134348506 | 13549.7939194384 | -1946.57323473727 | 8168.69214897064 | -1737.42320277854 |
| 13016.2129316318 | -6083.55258961582 | 14644.8962006979 | -2430.14448086803 | 9263.79443023006 | -2220.99444890930 |
| 12809.9390972627 | -6273.75375590940 | 14626.6987111848 | -2710.13417671444 | 9263.79443023006 | -2220.99444890930 |
| 13864.0008877415 | -6807.82318988943 | 15680.7605016635 | -3244.20361069447 | 10317.8562207088 | -2755.06388288933 |
| 13645.9497471408 | -6988.71401951158 | 15647.6136961404 | -3525.57361876616 | 10317.8562207088 | -2755.06388288933 |
| 14704.6024955725 | -7603.55483378853 | 16706.2664445722 | -4140.41443304311 | 11376.5089691406 | -3369.90469716628 |
| 14477.8451929186 | -7772.37146331932 | 16658.4881423268 | -4419.04530453361 | 11376.5089691406 | -3369.90469716628 |
| 15480.5837805564 | -8425.78310093937 | 17661.2267299645 | -5072.45694215365 | 12379.2475567783 | -4023.31633478632 |
| 15246.7628830765 | -8581.53483036608 | 17599.3231348423 | -5346.49846862123 | 12379.2475567783 | -4023.31633478632 |
| 16210.7397757543 | -9278.66626857911 | 18563.3000275202 | -6043.62990683426 | 13343.2244494562 | -4720.44777299936 |
| 15968.8682506153 | -9422.14902142367 | 18487.1053043813 | -6314.33915898999 | 13343.2244494562 | -4720.44777299936 |
| 16910.0511228137 | -10181.6489880057 | 19428.2881765797 | -7073.83912557206 | 14284.4073216546 | -5479.94773958143 |
| 16660.4122069618 | -10312.6075439745 | 19337.8314140447 | -7340.83595289565 | 14284.4073216546 | -5479.94773958143 |
| 17545.7404093842 | -11109.6330813827 | 20223.1596164671 | -8137.86149030381 | 15169.7355240771 | -6276.97327698959 |
| 17289.9295522786 | -11227.2033002086 | 20119.0001812016 | -8399.41982607817 | 15169.7355240771 | -6276.97327698959 |
| 18135.4492793431 | -12070.8433286325 | 20964.5199082661 | -9243.05985450208 | 16015.2552511416 | -7120.61330541350 |
| 17873.5239970640 | -12175.0012275984 | 20846.6829100405 | -9499.12266566989 | 16015.2552511416 | -7120.61330541350 |
| 18672.8222279590 | -13062.3988787217 | 21645.9811409355 | -10386.5203167932 | 16814.5534820366 | -8008.01095653682 |
| 18406.6311644305 | -13152.4534582296 | 21515.3287014893 | -10635.3122974910 | 16814.5534820366 | -8008.01095653682 |
| 19158.7905107145 | -14075.7784311221 | 22267.4880477733 | -11558.6372703834 | 17566.7128283206 | -8931.33592942925 |
| 18887.4193173700 | -14152.0384080027 | 22123.5709607863 | -11801.0125593967 | 17566.7128283206 | -8931.33592942925 |
| 19582.9409657737 | -15108.9947876247 | 22819.0926091901 | -12757.9689390187 | 18262.2344767244 | -9888.29230905125 |
| 19308.4780414177 | -15170.8460857144 | 22662.9996626493 | -12992.0425854671 | 18262.2344767244 | -9888.29230905125 |
| 19963.4902992084 | -16174.9044360333 | 23318.0119204399 | -13996.1009357861 | 18917.2467345151 | -10892.3506593702 |
| 19686.5127224603 | -16222.2877921881 | 23150.1201277291 | -14221.4320397243 | 18917.2467345151 | -10892.3506593702 |
| 20291.4677148483 | -17261.7628018084 | 23755.0751201170 | -15260.9070493446 | 19522.2017269030 | -11931.8256689905 |
| 20012.0658079132 | -17294.6638338185 | 23575.4399682056 | -15477.4229048934 | 19522.2017269030 | -11931.8256689905 |
| 20552.5995061277 | -18349.3905194348 | 24115.9736664200 | -16532.1495905096 | 20062.7354251174 | -12986.5523546067 |
| 20272.2730899435 | -18367.6390447669 | 23925.5638652743 | -16738.6926027737 | 20062.7354251174 | -12986.5523546067 |
| 20763.0633418223 | -19457.7844611481 | 24416.3541171532 | -17828.8380191549 | 20553.5256769963 | -14076.6977709879 |
| 20482.5813290752 | -19461.3952461946 | 24215.7366493339 | -18024.8885964280 | 20553.5256769963 | -14076.6977709879 |
| 20923.5644020039 | -20589.5579156873 | 24656.7197222627 | -19153.0512659207 | 20994.5087499251 | -15204.8604404805 |
| 20642.4047314779 | -20578.5019209101 | 24445.4965161734 | -19338.9484217896 | 20994.5087499251 | -15204.8604404805 |
| 21019.9882378930 | -21730.8330642626 | 24823.0800225885 | -20491.2795651421 | 21372.0922563401 | -16357.1915838330 |
| 20738.3567629492 | -21704.9368399589 | 24601.2805239715 | -20666.7557302627 | 21372.0922563401 | -16357.1915838330 |
| 21047.1642864343 | -22876.6682812412 | 24910.0880474565 | -21838.4871715450 | 21680.8997798252 | -17528.9230251153 |
| 20768.4990106334 | -22836.2316496800 | 24680.4086190758 | -22001.3884216564 | 21680.8997798252 | -17528.9230251153 |
| 21018.6033448263 | -24003.1624018841 | 24930.5129532686 | -23168.3191738606 | 21931.0041140180 | -18695.8537773194 |
| 20741.3583611160 | -23947.9717294034 | 24691.6868116296 | -23319.5569515195 | 21931.0041140180 | -18695.8537773194 |
| 20924.8538741767 | -25135.4326972971 | 24875.1823246904 | -24507.0179194132 | 22114.4996270788 | -19883.3147452131 |
| 20651.0929153192 | -25065.8277259954 | 24628.9382018613 | -24645.4149807863 | 22114.4996270788 | -19883.3147452131 |
| 20772.8972537967 | -26256.6888214929 | 24750.7425403388 | -25836.2760762837 | 22236.3039655563 | -21074.1758407106 |
| 20504.5493644387 | -26173.2959610361 | 24498.9093373853 | -25960.9556527515 | 22236.3039655563 | -21074.1758407106 |
| 20573.1611181558 | -27368.2413780582 | 24567.5210911024 | -27155.9010697737 | 22304.9157192734 | -22269.1212577327 |

d）differ theta

| k_1i_ | | k_2i_ | | O_i_ | |
| --- | --- | --- | --- | --- | --- |
| X | Y | X | X | Y | X |
| 5000 | -2000 | 5000 | 2000 | 0 | 0 |
| 4806.55782960332 | -2428.37431848532 | 5155.35175142676 | 1556.38951392671 | 0 | 0 |
| 5801.43560526964 | -2515.45785359650 | 6150.22952709308 | 1469.30597881553 | 994.877775666320 | -87.0835351111863 |
| 5571.79985877719 | -2924.65014140117 | 6266.14315053280 | 1014.62484178977 | 994.877775666320 | -87.0835351111863 |
| 6566.95415481489 | -3099.90472379960 | 7261.29744657050 | 839.370259391342 | 1990.03207170402 | -262.338117509611 |
| 6302.04916736693 | -3488.25016942776 | 7337.29414230003 | 375.461497075809 | 1990.03207170402 | -262.338117509611 |
| 7271.09707653065 | -3748.07790143780 | 8306.34205146374 | 115.633765065768 | 2959.07998086774 | -522.165849519652 |
| 6974.17836418167 | -4110.89612251453 | 8341.55141222946 | -351.868196644266 | 2959.07998086774 | -522.165849519652 |
| 7916.27527126315 | -4453.02274674195 | 9283.64831931094 | -693.994820871689 | 3901.17688794922 | -864.292473747075 |
| 7588.50511452178 | -4789.03584746226 | 9278.03068906396 | -1163.36303638734 | 3901.17688794922 | -864.292473747075 |
| 8489.37567758322 | -5208.71782514178 | 10178.9012521254 | -1583.04501406686 | 4802.04745101066 | -1283.97445142660 |
| 8132.61581448590 | -5515.67793539353 | 10132.2545307458 | -2051.36775810939 | 4802.04745101066 | -1283.97445142660 |
| 9003.37254991826 | -6019.50417599031 | 11003.0112661781 | -2555.19399870617 | 5672.80418644302 | -1787.80069202338 |
| 8621.07190584414 | -6294.20917935976 | 10915.6245952069 | -3017.77392809378 | 5672.80418644302 | -1787.80069202338 |
| 9453.65951052685 | -6878.75510867939 | 11748.2121998896 | -3602.31985741341 | 6505.39179112574 | -2372.34662134301 |
| 9048.84206703115 | -7119.01499968973 | 11620.7650104688 | -4055.48560159035 | 6505.39179112574 | -2372.34662134301 |
| 9808.33725401554 | -7758.13955032466 | 12380.2601974532 | -4694.61015222527 | 7264.88697811013 | -3011.47117197793 |
| 9385.14329058093 | -7961.67451824844 | 12214.1783397411 | -5133.85544860775 | 7264.88697811013 | -3011.47117197793 |
| 10098.9268703545 | -8674.98058908434 | 12927.9619195146 | -5847.16151944365 | 7978.67055788370 | -3724.77724281383 |
| 9660.35140892352 | -8840.63000801789 | 12724.6137708464 | -6269.58038241690 | 7978.67055788370 | -3724.77724281383 |
| 10304.0179335225 | -9604.89186537837 | 13368.2802954453 | -7033.84223977737 | 8622.33708248268 | -4489.03910017431 |
| 9852.14360817920 | -9731.89861663613 | 13128.6518193400 | -7437.45011161458 | 8622.33708248268 | -4489.03910017431 |
| 10427.4638334623 | -10552.0718187934 | 13703.9720446231 | -8257.62331377181 | 9197.65730776574 | -5309.21230233153 |
| 9964.92375241732 | -10639.4376449331 | 13429.2815212042 | -8639.88138169708 | 9197.65730776574 | -5309.21230233153 |
| 10463.7014466336 | -11507.4704678975 | 13928.0592154205 | -9507.91420466151 | 9696.43500198205 | -6177.24512529596 |
| 9995.14874764454 | -11554.1187613555 | 13620.9336702615 | -9864.83379577875 | 9696.43500198205 | -6177.24512529596 |
| 10415.8410400001 | -12462.4426449741 | 14041.6259626170 | -10773.1576793973 | 10117.1272943376 | -7085.56900891453 |
| 9945.16528889036 | -12467.9875237350 | 13704.6158523382 | -11101.7769027137 | 10117.1272943376 | -7085.56900891453 |
| 10280.9871929738 | -13397.5177593181 | 14040.4377564217 | -12031.3071382967 | 10452.9491984210 | -8015.09924449762 |
| 9812.36809148681 | -13362.0288162649 | 13676.6177566874 | -12328.7938357320 | 10452.9491984210 | -8015.09924449762 |
| 10067.7933748869 | -14317.0546887175 | 13932.0430400876 | -13283.8197081846 | 10708.3744818211 | -8970.12511695027 |
| 9604.59618534923 | -14240.9574489675 | 13544.1435305947 | -13548.1611480468 | 10708.3744818211 | -8970.12511695027 |
| 9778.62482209949 | -15221.2944570231 | 13718.1721673450 | -14528.4981561024 | 10882.4031185714 | -9950.46212500595 |
| 9321.63948399069 | -15104.4913136088 | 13306.6978580839 | -14759.0788716361 | 10882.4031185714 | -9950.46212500595 |
| 9402.69464099926 | -16099.0083652542 | 13387.7530150925 | -15753.5959232815 | 10963.4582755799 | -10944.9791766514 |
| 8959.80745431369 | -15943.5173015874 | 12959.8063877223 | -15946.4383852369 | 10963.4582755799 | -10944.9791766514 |
| 8961.16991704067 | -16947.4090390720 | 12961.1688504493 | -16950.3301227216 | 10964.8207383069 | -11948.8709141360 |
| 8533.44507917739 | -16753.9112265780 | 12517.9903040697 | -17105.1936639247 | 10964.8207383069 | -11948.8709141360 |
| 8445.95021433409 | -17767.9260124742 | 12430.4954392264 | -18119.2084498209 | 10877.3258734636 | -12962.8857000322 |
| 8035.28506120420 | -17537.0308684164 | 11973.8791945062 | -18235.2258649031 | 10877.3258734636 | -12962.8857000322 |
| 7859.06264384088 | -18508.3419473965 | 11797.6567771429 | -19206.5369438832 | 10701.1034561003 | -13934.1967790124 |
| 7470.44073097576 | -18242.6557095919 | 11333.0090210650 | -19282.1585736559 | 10701.1034561003 | -13934.1967790124 |
| 7205.61387688797 | -19216.3018027542 | 11068.1821669772 | -20255.8046668181 | 10436.2766020125 | -14907.8428721746 |
| 6842.36735767721 | -18918.3062298780 | 10599.6273959099 | -20290.5296100531 | 10436.2766020125 | -14907.8428721746 |
| 6499.94749944962 | -19858.5637608601 | 10257.2075376824 | -21230.7871410353 | 10093.8567437849 | -15848.1004031568 |
| 6163.98864192909 | -19529.9663298325 | 9787.30806773468 | -21224.5330409950 | 10093.8567437849 | -15848.1004031568 |
| 5731.38013105773 | -20456.1894485517 | 9354.69955686332 | -22150.7561597141 | 9661.24823291356 | -16774.3235218759 |
| 5426.71208140082 | -20101.2896445666 | 8889.31938022551 | -22103.8756462470 | 9661.24823291356 | -16774.3235218759 |
| 4937.18633211897 | -20967.4558048194 | 8399.79363094366 | -22970.0418064997 | 9171.72248363172 | -17640.4896821287 |
